# Poly[[(2,2′-bipyridine)(*μ*_3_-7-oxabicyclo­[2.2.1]heptane-2,3-dicarboxyl­ato)cadmium] monohydrate]

**DOI:** 10.1107/S1600536811036634

**Published:** 2011-09-14

**Authors:** Ling-Ling Zhang, Shi-Kun Li, Jie Feng, Qiu-Yue Lin

**Affiliations:** a College of Chemistry and Life Science, Zhejiang Normal University, Jinhua 321004, Zhejiang, People’s Republic of China; b Zhejiang Key Laboratory for Reactive Chemistry on Solid Surfaces, Institute of Physical Chemistry, Zhejiang Normal University, Jinhua, Zhejiang 321004, People’s Republic of China

## Abstract

The title compound, {[Cd(C_8_H_8_O_5_)(C_10_H_8_N_2_)]·H_2_O}_*n*_, was obtained by the reaction of cadmium acetate with 2,2′-bi­pyridine and 7-oxabicyclo­(2.2.1)heptane-2,3-dicarb­oxy­lic anhydride. The Cd^II^ atom is seven-coordinated in a distorted penta­gonal–bipyramidal configuration, defined by five O atoms from the carboxyl­ate groups of three 7-oxabicyclo­[2.2.1]heptane-2,3-dicarboxyl­ato ligands and two N atoms from the 2,2′-bipyridine ligand. Two O atoms link two Cd^II^ atoms, forming a dinuclear center: the Cd—O—Cd bridging angle is 110.19 (6)°. The polymeric structure extends along [100] and is linked by inter­molecular O—H⋯O hydrogen bonds involving the solvent water molecule. Extensive π–π stacking exists between 2,2-bypiridine ligands along [010] with centroid-centroid distance of 3.650 (2) Å

## Related literature

For background to the applications of norcantharidin [systematic name: 7-oxabicyclo­[2.2.1]heptane-2,3-dicarb­oxy­lic anhydride], see: Wang *et al.* (1989 ▶). F or related structures, see: Yin *et al.* (2003 ▶); Wang *et al.* (2009 ▶).
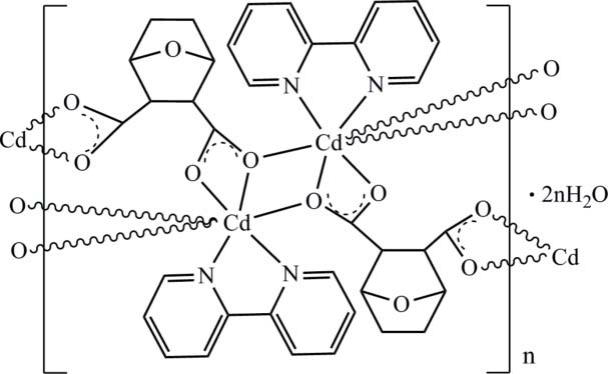


## Experimental

### 

#### Crystal data

[Cd(C_8_H_8_O_5_)(C_10_H_8_N_2_)]·H_2_O
*M* *_r_* = 470.75
Triclinic, 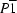

*a* = 8.2599 (1) Å
*b* = 10.5950 (2) Å
*c* = 11.1097 (2) Å
α = 111.784 (1)°
β = 94.066 (1)°
γ = 102.749 (1)°
*V* = 867.94 (2) Å^3^
*Z* = 2
Mo *K*α radiation
μ = 1.30 mm^−1^
*T* = 296 K
0.33 × 0.14 × 0.07 mm

#### Data collection

Bruker SMART APEXII CCD diffractometer
Absorption correction: multi-scan (*SADABS*; Sheldrick, 1996 ▶) *T*_min_ = 0.803, *T*_max_ = 0.918
13190 measured reflections
3972 independent reflections
3699 reflections with *I* > 2σ(*I*)
*R*_int_ = 0.021

#### Refinement

*R*[*F* ^2^ > 2σ(*F* ^2^)] = 0.022
*wR*(*F* ^2^) = 0.059
*S* = 0.95
3972 reflections
250 parameters
3 restraints
H atoms treated by a mixture of independent and constrained refinement
Δρ_max_ = 0.34 e Å^−3^
Δρ_min_ = −0.56 e Å^−3^

### 

Data collection: *APEX2* (Bruker, 2004 ▶); cell refinement: *SAINT* (Bruker, 2001 ▶); data reduction: *SAINT*; program(s) used to solve structure: *SHELXS97* (Sheldrick, 2008 ▶); program(s) used to refine structure: *SHELXL97* (Sheldrick, 2008 ▶); molecular graphics: *SHELXTL* (Sheldrick, 2008 ▶); software used to prepare material for publication: *SHELXTL*.

## Supplementary Material

Crystal structure: contains datablock(s) global. DOI: 10.1107/S1600536811036634/zb2016sup1.cif

Structure factors: contains datablock(s) I. DOI: 10.1107/S1600536811036634/zb2016Isup2.hkl

Additional supplementary materials:  crystallographic information; 3D view; checkCIF report

## Figures and Tables

**Table 1:** Hydrogen-bond geometry (Å, °)

*D*—H⋯*A*	*D*—H	H⋯*A*	*D*⋯*A*	*D*—H⋯*A*
O1*W*—H1*WA*⋯O3^i^	0.92 (2)	2.19 (4)	2.980 (4)	144 (5)
O1*W*—H1*WA*⋯O1*W*^ii^	0.92 (2)	2.38 (6)	2.833 (8)	110 (5)

Symmetry codes: (i) 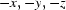; (ii) 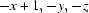.

**Fig. 1.**
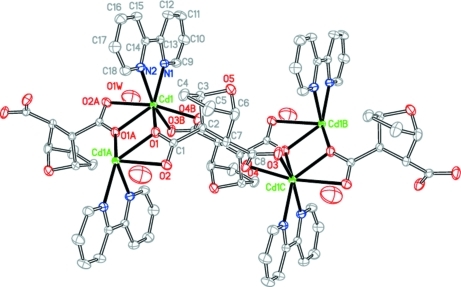
A view of the molecule of (I) showing the atom-labelling scheme with displacement ellipsoids drawn at the 30% probability, hydrogen atoms and water molecules were omitted.

## supplementary crystallographic information

### Comment

7-oxabicyclo(2,2,1)heptane-2,3-dicarboxylic anhydride (norcantharidin) is a variety of pharmacologically important compound such as protein kinase inhibitors and antitumor properties (Wang, 1989). Demethylcantharate is the acid radical of norcantharidin. A copper complex of 2,2'-bipyridine and demethylcantharate was reported (Yin *et al.*, 2003); and a similar cadmium complex of demethylcantharate (Wang *et al.*, 2009) has been reported.

Cadmium acetate can react with 2,2'-bipyridine and norcantharidin to form the title compound. X-ray crystallography measurement confirmed the molecular structure and the atom connectivity for the title compound (Fig. 1). The cadmium atom is seven-coordinated in a distorted pentagonal bipyramidal configuration, defined by five oxygen atoms (O1,O2,O1A,O3B,O4B) from carboxylate groups of three demethylcantharates and two nitrogen atoms (N1,N2) from 2,2'-bipyridine. O1 and O1A link two cadmium atoms(Cd1,Cd1A) to form a dinuclear center, and the The angle of the bridging O1(Cd1—O1—Cd1A)), is 110.19 (6)°.Each demethylcantharate acts as a four-coordinated bridging linker that connects two cadmium centers.

It showed that the polymeric molecules grow in [100] direction,and are linked by H-bonds(O1W—H1WA···O3; O1W—H1WA···O1W) to form a plane. Extensive pi-stacking is observed between 2,2-bypiridine ligands propagated along [010], linking the planes with the distance between planes of 3.4738 Å.

### Experimental

A mixture of 0.5 mmol norcantharidin, 0.5 mmol 2,2'-bipyridine,0.5 mmol cadmium acetate and 10 mL distilled water was sealed in a 25 mL Teflon-lined stainless vessel and heated at 433 K for 3 d, then cooled slowly to room temperature. The solution was filtered and block shaped colorless transparent crystals were obtained.

### Refinement

The structure was solved by direct methods and successive Fourier difference synthesis. The H atoms bonded to C atoms were positioned geometrically and refined using a riding model [aromatic C—H = 0.93 Å, aliphatic of tertiary carbon C—H = 0.98 Å, aliphatic of secondary carbon C—H = 0.97 Å, *U*_iso_(H) = 1.2*U*_eq_(C)]. The H atoms bonded to O atoms were located in a difference Fourier maps and refined with O—H distance restraints of 0.85 (2) Å and *U*_iso_(H) = 1.5*U*_eq_(O).

### Crystal data

**Table:**

[Cd(C_8_H_8_O_5_)(C_10_H_8_N_2_)]·H_2_O	*Z* = 2
*M**_r_* = 470.75	*F*(000) = 472
Triclinic, *P*1	*D*_x_ = 1.801 Mg m^−^^3^
Hall symbol: -P 1	Mo *K*α radiation, λ = 0.71073 Å
*a* = 8.2599 (1) Å	Cell parameters from 7860 reflections
*b* = 10.5950 (2) Å	θ = 2.0–27.6°
*c* = 11.1097 (2) Å	µ = 1.30 mm^−^^1^
α = 111.784 (1)°	*T* = 296 K
β = 94.066 (1)°	Block, colourless
γ = 102.749 (1)°	0.33 × 0.14 × 0.07 mm
*V* = 867.94 (2) Å^3^	

### Data collection

**Table:**

Bruker SMART APEXII CCD diffractometer	3972 independent reflections
Radiation source: fine-focus sealed tube	3699 reflections with *I* > 2σ(*I*)
graphite	*R*_int_ = 0.021
ω scans	θ_max_ = 27.6°, θ_min_ = 2.0°
Absorption correction: multi-scan (*SADABS*; Sheldrick, 1996)	*h* = −10→10
*T*_min_ = 0.803, *T*_max_ = 0.918	*k* = −13→13
13190 measured reflections	*l* = −14→14

### Refinement

**Table:**

Refinement on *F*^2^	Primary atom site location: structure-invariant direct methods
Least-squares matrix: full	Secondary atom site location: difference Fourier map
*R*[*F*^2^ > 2σ(*F*^2^)] = 0.022	Hydrogen site location: inferred from neighbouring sites
*wR*(*F*^2^) = 0.059	H atoms treated by a mixture of independent and constrained refinement
*S* = 0.95	*w* = 1/[σ^2^(*F*_o_^2^) + (0.0358*P*)^2^ + 0.509*P*] where *P* = (*F*_o_^2^ + 2*F*_c_^2^)/3
3972 reflections	(Δ/σ)_max_ < 0.001
250 parameters	Δρ_max_ = 0.34 e Å^−^^3^
3 restraints	Δρ_min_ = −0.56 e Å^−^^3^

### Special details

**Table:**

Geometry. All e.s.d.'s (except the e.s.d. in the dihedral angle between two l.s. planes) are estimated using the full covariance matrix. The cell e.s.d.'s are taken into account individually in the estimation of e.s.d.'s in distances, angles and torsion angles; correlations between e.s.d.'s in cell parameters are only used when they are defined by crystal symmetry. An approximate (isotropic) treatment of cell e.s.d.'s is used for estimating e.s.d.'s involving l.s. planes.
Refinement. Refinement of *F*^2^ against ALL reflections. The weighted *R*-factor *wR* and goodness of fit *S* are based on *F*^2^, conventional *R*-factors *R* are based on *F*, with *F* set to zero for negative *F*^2^. The threshold expression of *F*^2^ > σ(*F*^2^) is used only for calculating *R*-factors(gt) *etc*. and is not relevant to the choice of reflections for refinement. *R*-factors based on *F*^2^ are statistically about twice as large as those based on *F*, and *R*- factors based on ALL data will be even larger.

### Fractional atomic coordinates and isotropic or equivalent isotropic displacement parameters (Å^2^)

**Table:**

	*x*	*y*	*z*	*U*_iso_*/*U*_eq_	
Cd1	0.622884 (17)	0.135016 (14)	0.438680 (14)	0.02838 (6)	
N1	0.7226 (2)	0.34613 (18)	0.41261 (17)	0.0312 (4)	
N2	0.5919 (2)	0.32243 (19)	0.62096 (18)	0.0329 (4)	
O1W	0.4578 (5)	0.1298 (4)	0.0551 (3)	0.1174 (12)	
H1WA	0.411 (7)	0.057 (5)	−0.024 (3)	0.176*	
H1WB	0.400 (8)	0.088 (6)	0.104 (5)	0.176*	
O1	0.37311 (19)	−0.02140 (17)	0.40538 (16)	0.0371 (3)	
O2	0.3303 (2)	0.11060 (18)	0.30183 (19)	0.0449 (4)	
O3	−0.3040 (2)	−0.0038 (2)	0.22846 (18)	0.0500 (5)	
O4	−0.1199 (2)	0.09023 (19)	0.40964 (15)	0.0432 (4)	
O5	−0.0940 (2)	−0.27932 (18)	0.14335 (18)	0.0474 (4)	
C1	0.2765 (2)	0.0124 (2)	0.33481 (19)	0.0274 (4)	
C2	0.0974 (2)	−0.0774 (2)	0.29330 (19)	0.0267 (4)	
H2A	0.0520	−0.0788	0.3721	0.032*	
C3	0.0822 (3)	−0.2314 (2)	0.1986 (2)	0.0361 (5)	
H3A	0.1177	−0.2882	0.2426	0.043*	
C4	0.1675 (3)	−0.2356 (3)	0.0796 (2)	0.0452 (6)	
H4A	0.1784	−0.3293	0.0290	0.054*	
H4B	0.2776	−0.1685	0.1058	0.054*	
C5	0.0417 (4)	−0.1935 (4)	0.0024 (3)	0.0565 (7)	
H5A	0.0937	−0.1068	−0.0067	0.068*	
H5B	−0.0049	−0.2675	−0.0841	0.068*	
C6	−0.0928 (3)	−0.1738 (3)	0.0910 (2)	0.0439 (6)	
H6A	−0.2029	−0.1827	0.0448	0.053*	
C7	−0.0255 (3)	−0.0357 (2)	0.21353 (19)	0.0296 (4)	
H7A	0.0382	0.0371	0.1878	0.036*	
C8	−0.1600 (3)	0.0185 (2)	0.2884 (2)	0.0305 (4)	
C9	0.7903 (3)	0.3532 (3)	0.3088 (2)	0.0402 (5)	
H9A	0.7896	0.2695	0.2400	0.048*	
C10	0.8609 (3)	0.4784 (3)	0.2992 (3)	0.0465 (6)	
H10A	0.9063	0.4794	0.2252	0.056*	
C11	0.8631 (4)	0.6025 (3)	0.4012 (3)	0.0477 (6)	
H11A	0.9115	0.6889	0.3979	0.057*	
C12	0.7923 (3)	0.5967 (2)	0.5088 (2)	0.0407 (5)	
H12A	0.7914	0.6793	0.5784	0.049*	
C13	0.7229 (3)	0.4670 (2)	0.5117 (2)	0.0295 (4)	
C14	0.6425 (3)	0.4529 (2)	0.6236 (2)	0.0295 (4)	
C15	0.6177 (3)	0.5688 (2)	0.7256 (2)	0.0393 (5)	
H15A	0.6548	0.6591	0.7273	0.047*	
C16	0.5372 (4)	0.5476 (3)	0.8235 (2)	0.0476 (6)	
H16A	0.5199	0.6237	0.8924	0.057*	
C17	0.4825 (3)	0.4128 (3)	0.8190 (2)	0.0463 (6)	
H17A	0.4261	0.3964	0.8835	0.056*	
C18	0.5134 (3)	0.3037 (3)	0.7171 (2)	0.0410 (5)	
H18A	0.4784	0.2129	0.7145	0.049*	

### Atomic displacement parameters (Å^2^)

**Table:**

	*U*^11^	*U*^22^	*U*^33^	*U*^12^	*U*^13^	*U*^23^
Cd1	0.02580 (9)	0.02276 (9)	0.03633 (9)	0.00709 (6)	0.00848 (6)	0.01055 (6)
N1	0.0303 (9)	0.0274 (9)	0.0358 (9)	0.0080 (7)	0.0086 (7)	0.0118 (7)
N2	0.0360 (10)	0.0287 (9)	0.0367 (9)	0.0109 (7)	0.0097 (8)	0.0139 (7)
O1W	0.152 (3)	0.093 (2)	0.087 (2)	0.012 (2)	−0.004 (2)	0.0295 (18)
O1	0.0269 (8)	0.0421 (9)	0.0458 (9)	0.0071 (6)	0.0014 (6)	0.0233 (7)
O2	0.0348 (9)	0.0379 (9)	0.0687 (11)	0.0044 (7)	0.0074 (8)	0.0317 (8)
O3	0.0282 (8)	0.0570 (11)	0.0526 (10)	0.0190 (8)	0.0038 (7)	0.0047 (8)
O4	0.0451 (10)	0.0547 (10)	0.0328 (8)	0.0276 (8)	0.0114 (7)	0.0118 (7)
O5	0.0317 (9)	0.0380 (9)	0.0558 (10)	0.0016 (7)	0.0018 (7)	0.0058 (8)
C1	0.0246 (9)	0.0265 (10)	0.0305 (9)	0.0095 (7)	0.0078 (8)	0.0086 (8)
C2	0.0238 (9)	0.0296 (10)	0.0299 (9)	0.0072 (8)	0.0074 (7)	0.0147 (8)
C3	0.0306 (11)	0.0291 (11)	0.0454 (12)	0.0065 (8)	0.0051 (9)	0.0122 (9)
C4	0.0414 (13)	0.0494 (14)	0.0414 (12)	0.0219 (11)	0.0124 (10)	0.0078 (11)
C5	0.0595 (17)	0.078 (2)	0.0342 (12)	0.0364 (15)	0.0134 (12)	0.0131 (13)
C6	0.0334 (12)	0.0545 (15)	0.0339 (11)	0.0177 (11)	−0.0002 (9)	0.0042 (10)
C7	0.0254 (10)	0.0361 (11)	0.0298 (9)	0.0104 (8)	0.0068 (8)	0.0144 (8)
C8	0.0291 (10)	0.0288 (10)	0.0385 (11)	0.0101 (8)	0.0113 (8)	0.0163 (9)
C9	0.0457 (13)	0.0340 (12)	0.0401 (12)	0.0102 (10)	0.0141 (10)	0.0130 (10)
C10	0.0549 (15)	0.0436 (14)	0.0423 (13)	0.0054 (11)	0.0162 (11)	0.0212 (11)
C11	0.0589 (16)	0.0339 (13)	0.0485 (14)	−0.0002 (11)	0.0099 (12)	0.0214 (11)
C12	0.0507 (14)	0.0261 (11)	0.0401 (12)	0.0038 (10)	0.0052 (10)	0.0115 (9)
C13	0.0264 (10)	0.0273 (10)	0.0334 (10)	0.0066 (8)	0.0009 (8)	0.0117 (8)
C14	0.0283 (10)	0.0273 (10)	0.0324 (10)	0.0087 (8)	0.0017 (8)	0.0110 (8)
C15	0.0475 (13)	0.0276 (11)	0.0396 (12)	0.0107 (9)	0.0082 (10)	0.0091 (9)
C16	0.0561 (16)	0.0411 (14)	0.0384 (12)	0.0153 (11)	0.0118 (11)	0.0058 (10)
C17	0.0537 (15)	0.0497 (15)	0.0385 (12)	0.0149 (12)	0.0174 (11)	0.0186 (11)
C18	0.0489 (14)	0.0344 (12)	0.0436 (12)	0.0112 (10)	0.0156 (11)	0.0183 (10)

### Geometric parameters (Å, °)

**Table:**

Cd1—O1	2.2511 (15)	C3—H3A	0.9800
Cd1—O4^i^	2.2963 (16)	C4—C5	1.541 (4)
Cd1—N2	2.3285 (18)	C4—H4A	0.9700
Cd1—N1	2.3382 (17)	C4—H4B	0.9700
Cd1—O1^ii^	2.4513 (15)	C5—C6	1.534 (3)
Cd1—O3^i^	2.4546 (17)	C5—H5A	0.9700
Cd1—O2	2.6704 (17)	C5—H5B	0.9700
Cd1—C8^i^	2.720 (2)	C6—C7	1.535 (3)
Cd1—C1	2.826 (2)	C6—H6A	0.9800
N1—C9	1.336 (3)	C7—C8	1.521 (3)
N1—C13	1.343 (3)	C7—H7A	0.9800
N2—C18	1.341 (3)	C8—Cd1^iii^	2.720 (2)
N2—C14	1.341 (3)	C9—C10	1.373 (3)
O1W—H1WA	0.915 (19)	C9—H9A	0.9300
O1W—H1WB	0.922 (19)	C10—C11	1.377 (4)
O1—C1	1.275 (2)	C10—H10A	0.9300
O1—Cd1^ii^	2.4513 (15)	C11—C12	1.383 (3)
O2—C1	1.232 (3)	C11—H11A	0.9300
O3—C8	1.249 (3)	C12—C13	1.381 (3)
O3—Cd1^iii^	2.4546 (17)	C12—H12A	0.9300
O4—C8	1.252 (3)	C13—C14	1.490 (3)
O4—Cd1^iii^	2.2963 (16)	C14—C15	1.395 (3)
O5—C3	1.438 (3)	C15—C16	1.376 (4)
O5—C6	1.438 (3)	C15—H15A	0.9300
C1—C2	1.504 (3)	C16—C17	1.380 (4)
C2—C7	1.540 (3)	C16—H16A	0.9300
C2—C3	1.550 (3)	C17—C18	1.373 (3)
C2—H2A	0.9800	C17—H17A	0.9300
C3—C4	1.533 (3)	C18—H18A	0.9300
			
O1—Cd1—O4^i^	127.85 (6)	C7—C2—H2A	108.5
O1—Cd1—N2	99.82 (6)	C3—C2—H2A	108.5
O4^i^—Cd1—N2	121.75 (6)	O5—C3—C4	102.84 (19)
O1—Cd1—N1	137.35 (6)	O5—C3—C2	101.22 (16)
O4^i^—Cd1—N1	88.94 (6)	C4—C3—C2	110.98 (19)
N2—Cd1—N1	70.49 (6)	O5—C3—H3A	113.6
O1—Cd1—O1^ii^	69.81 (6)	C4—C3—H3A	113.6
O4^i^—Cd1—O1^ii^	84.38 (6)	C2—C3—H3A	113.6
N2—Cd1—O1^ii^	83.14 (6)	C3—C4—C5	101.08 (19)
N1—Cd1—O1^ii^	143.93 (6)	C3—C4—H4A	111.6
O1—Cd1—O3^i^	93.72 (6)	C5—C4—H4A	111.6
O4^i^—Cd1—O3^i^	54.58 (6)	C3—C4—H4B	111.6
N2—Cd1—O3^i^	162.62 (7)	C5—C4—H4B	111.6
N1—Cd1—O3^i^	92.16 (7)	H4A—C4—H4B	109.4
O1^ii^—Cd1—O3^i^	111.97 (6)	C6—C5—C4	101.4 (2)
O1—Cd1—O2	51.77 (5)	C6—C5—H5A	111.5
O4^i^—Cd1—O2	141.20 (6)	C4—C5—H5A	111.5
N2—Cd1—O2	92.73 (6)	C6—C5—H5B	111.5
N1—Cd1—O2	86.47 (5)	C4—C5—H5B	111.5
O1^ii^—Cd1—O2	119.82 (5)	H5A—C5—H5B	109.3
O3^i^—Cd1—O2	87.10 (6)	O5—C6—C5	102.7 (2)
O1—Cd1—C8^i^	112.42 (6)	O5—C6—C7	102.64 (18)
O4^i^—Cd1—C8^i^	27.27 (6)	C5—C6—C7	109.6 (2)
N2—Cd1—C8^i^	146.41 (7)	O5—C6—H6A	113.6
N1—Cd1—C8^i^	90.67 (6)	C5—C6—H6A	113.6
O1^ii^—Cd1—C8^i^	98.88 (6)	C7—C6—H6A	113.6
O3^i^—Cd1—C8^i^	27.32 (6)	C8—C7—C6	114.70 (18)
O2—Cd1—C8^i^	114.24 (6)	C8—C7—C2	113.22 (16)
O1—Cd1—C1	26.07 (5)	C6—C7—C2	101.20 (17)
O4^i^—Cd1—C1	140.84 (6)	C8—C7—H7A	109.1
N2—Cd1—C1	96.88 (6)	C6—C7—H7A	109.1
N1—Cd1—C1	111.81 (6)	C2—C7—H7A	109.1
O1^ii^—Cd1—C1	95.02 (5)	O3—C8—O4	121.56 (19)
O3^i^—Cd1—C1	90.48 (6)	O3—C8—C7	120.25 (19)
O2—Cd1—C1	25.70 (5)	O4—C8—C7	118.12 (18)
C8^i^—Cd1—C1	116.18 (6)	O3—C8—Cd1^iii^	64.41 (12)
O1—Cd1—Cd1^ii^	36.61 (4)	O4—C8—Cd1^iii^	57.15 (11)
O4^i^—Cd1—Cd1^ii^	107.21 (5)	C7—C8—Cd1^iii^	174.70 (15)
N2—Cd1—Cd1^ii^	91.35 (5)	N1—C9—C10	123.0 (2)
N1—Cd1—Cd1^ii^	160.51 (4)	N1—C9—H9A	118.5
O1^ii^—Cd1—Cd1^ii^	33.21 (4)	C10—C9—H9A	118.5
O3^i^—Cd1—Cd1^ii^	105.99 (5)	C9—C10—C11	118.7 (2)
O2—Cd1—Cd1^ii^	87.42 (3)	C9—C10—H10A	120.7
C8^i^—Cd1—Cd1^ii^	108.70 (4)	C11—C10—H10A	120.7
C1—Cd1—Cd1^ii^	62.06 (4)	C10—C11—C12	119.0 (2)
C9—N1—C13	118.48 (19)	C10—C11—H11A	120.5
C9—N1—Cd1	123.34 (14)	C12—C11—H11A	120.5
C13—N1—Cd1	117.99 (14)	C13—C12—C11	119.3 (2)
C18—N2—C14	119.07 (19)	C13—C12—H12A	120.4
C18—N2—Cd1	122.58 (15)	C11—C12—H12A	120.4
C14—N2—Cd1	118.11 (14)	N1—C13—C12	121.7 (2)
H1WA—O1W—H1WB	95 (2)	N1—C13—C14	116.21 (18)
C1—O1—Cd1	103.02 (13)	C12—C13—C14	122.13 (19)
C1—O1—Cd1^ii^	143.44 (13)	N2—C14—C15	121.1 (2)
Cd1—O1—Cd1^ii^	110.19 (6)	N2—C14—C13	116.89 (18)
C1—O2—Cd1	84.22 (12)	C15—C14—C13	121.98 (19)
C8—O3—Cd1^iii^	88.27 (13)	C16—C15—C14	119.0 (2)
C8—O4—Cd1^iii^	95.58 (13)	C16—C15—H15A	120.5
C3—O5—C6	96.14 (17)	C14—C15—H15A	120.5
O2—C1—O1	120.98 (19)	C15—C16—C17	119.7 (2)
O2—C1—C2	123.43 (18)	C15—C16—H16A	120.2
O1—C1—C2	115.57 (17)	C17—C16—H16A	120.2
O2—C1—Cd1	70.08 (12)	C18—C17—C16	118.4 (2)
O1—C1—Cd1	50.91 (10)	C18—C17—H17A	120.8
C2—C1—Cd1	166.44 (14)	C16—C17—H17A	120.8
C1—C2—C7	117.17 (16)	N2—C18—C17	122.8 (2)
C1—C2—C3	112.65 (16)	N2—C18—H18A	118.6
C7—C2—C3	101.19 (16)	C17—C18—H18A	118.6
C1—C2—H2A	108.5		
			
O1—Cd1—N1—C9	−98.43 (19)	N1—Cd1—C1—O1	−170.00 (12)
O4^i^—Cd1—N1—C9	53.92 (18)	O1^ii^—Cd1—C1—O1	−14.62 (16)
N2—Cd1—N1—C9	178.32 (19)	O3^i^—Cd1—C1—O1	97.48 (13)
O1^ii^—Cd1—N1—C9	132.87 (17)	O2—Cd1—C1—O1	179.8 (2)
O3^i^—Cd1—N1—C9	−0.57 (18)	C8^i^—Cd1—C1—O1	87.84 (13)
O2—Cd1—N1—C9	−87.53 (18)	Cd1^ii^—Cd1—C1—O1	−10.42 (11)
C8^i^—Cd1—N1—C9	26.71 (18)	O1—Cd1—C1—C2	−4.7 (5)
C1—Cd1—N1—C9	−91.96 (18)	O4^i^—Cd1—C1—C2	68.0 (6)
Cd1^ii^—Cd1—N1—C9	−159.50 (14)	N2—Cd1—C1—C2	−103.0 (6)
O1—Cd1—N1—C13	86.62 (17)	N1—Cd1—C1—C2	−174.7 (5)
O4^i^—Cd1—N1—C13	−121.02 (15)	O1^ii^—Cd1—C1—C2	−19.3 (6)
N2—Cd1—N1—C13	3.37 (14)	O3^i^—Cd1—C1—C2	92.8 (6)
O1^ii^—Cd1—N1—C13	−42.07 (19)	O2—Cd1—C1—C2	175.1 (6)
O3^i^—Cd1—N1—C13	−175.52 (15)	C8^i^—Cd1—C1—C2	83.2 (6)
O2—Cd1—N1—C13	97.52 (15)	Cd1^ii^—Cd1—C1—C2	−15.1 (5)
C8^i^—Cd1—N1—C13	−148.23 (15)	O2—C1—C2—C7	−4.4 (3)
C1—Cd1—N1—C13	93.09 (15)	O1—C1—C2—C7	177.14 (17)
Cd1^ii^—Cd1—N1—C13	25.6 (2)	Cd1—C1—C2—C7	−178.8 (5)
O1—Cd1—N2—C18	37.44 (19)	O2—C1—C2—C3	112.4 (2)
O4^i^—Cd1—N2—C18	−109.65 (19)	O1—C1—C2—C3	−66.1 (2)
N1—Cd1—N2—C18	174.4 (2)	Cd1—C1—C2—C3	−62.1 (6)
O1^ii^—Cd1—N2—C18	−30.63 (18)	C6—O5—C3—C4	−56.7 (2)
O3^i^—Cd1—N2—C18	178.09 (18)	C6—O5—C3—C2	58.15 (19)
O2—Cd1—N2—C18	89.09 (18)	C1—C2—C3—O5	−162.72 (17)
C8^i^—Cd1—N2—C18	−126.37 (18)	C7—C2—C3—O5	−36.8 (2)
C1—Cd1—N2—C18	63.63 (19)	C1—C2—C3—C4	−54.1 (2)
Cd1^ii^—Cd1—N2—C18	1.61 (18)	C7—C2—C3—C4	71.8 (2)
O1—Cd1—N2—C14	−136.90 (15)	O5—C3—C4—C5	34.9 (2)
O4^i^—Cd1—N2—C14	76.02 (16)	C2—C3—C4—C5	−72.7 (2)
N1—Cd1—N2—C14	0.04 (14)	C3—C4—C5—C6	−0.3 (3)
O1^ii^—Cd1—N2—C14	155.04 (16)	C3—O5—C6—C5	56.3 (2)
O3^i^—Cd1—N2—C14	3.8 (3)	C3—O5—C6—C7	−57.48 (19)
O2—Cd1—N2—C14	−85.25 (15)	C4—C5—C6—O5	−34.4 (3)
C8^i^—Cd1—N2—C14	59.3 (2)	C4—C5—C6—C7	74.2 (3)
C1—Cd1—N2—C14	−110.71 (15)	O5—C6—C7—C8	−88.6 (2)
Cd1^ii^—Cd1—N2—C14	−172.72 (15)	C5—C6—C7—C8	162.76 (19)
O4^i^—Cd1—O1—C1	−130.25 (12)	O5—C6—C7—C2	33.6 (2)
N2—Cd1—O1—C1	85.57 (13)	C5—C6—C7—C2	−75.0 (2)
N1—Cd1—O1—C1	13.77 (17)	C1—C2—C7—C8	−111.9 (2)
O1^ii^—Cd1—O1—C1	164.46 (17)	C3—C2—C7—C8	125.18 (18)
O3^i^—Cd1—O1—C1	−83.48 (13)	C1—C2—C7—C6	124.80 (19)
O2—Cd1—O1—C1	−0.13 (11)	C3—C2—C7—C6	1.9 (2)
C8^i^—Cd1—O1—C1	−104.04 (13)	Cd1^iii^—O3—C8—O4	−0.2 (2)
Cd1^ii^—Cd1—O1—C1	164.46 (17)	Cd1^iii^—O3—C8—C7	−177.16 (17)
O4^i^—Cd1—O1—Cd1^ii^	65.30 (9)	Cd1^iii^—O4—C8—O3	0.2 (2)
N2—Cd1—O1—Cd1^ii^	−78.88 (7)	Cd1^iii^—O4—C8—C7	177.23 (16)
N1—Cd1—O1—Cd1^ii^	−150.68 (7)	C6—C7—C8—O3	−31.7 (3)
O1^ii^—Cd1—O1—Cd1^ii^	0.0	C2—C7—C8—O3	−147.1 (2)
O3^i^—Cd1—O1—Cd1^ii^	112.06 (7)	C6—C7—C8—O4	151.3 (2)
O2—Cd1—O1—Cd1^ii^	−164.59 (10)	C2—C7—C8—O4	35.8 (3)
C8^i^—Cd1—O1—Cd1^ii^	91.51 (8)	C13—N1—C9—C10	0.4 (4)
C1—Cd1—O1—Cd1^ii^	−164.46 (17)	Cd1—N1—C9—C10	−174.6 (2)
O1—Cd1—O2—C1	0.13 (12)	N1—C9—C10—C11	0.4 (4)
O4^i^—Cd1—O2—C1	105.62 (14)	C9—C10—C11—C12	−0.9 (4)
N2—Cd1—O2—C1	−100.23 (13)	C10—C11—C12—C13	0.7 (4)
N1—Cd1—O2—C1	−170.48 (13)	C9—N1—C13—C12	−0.6 (3)
O1^ii^—Cd1—O2—C1	−16.58 (15)	Cd1—N1—C13—C12	174.62 (17)
O3^i^—Cd1—O2—C1	97.17 (13)	C9—N1—C13—C14	178.72 (19)
C8^i^—Cd1—O2—C1	100.38 (13)	Cd1—N1—C13—C14	−6.1 (2)
Cd1^ii^—Cd1—O2—C1	−9.00 (12)	C11—C12—C13—N1	0.1 (4)
Cd1—O2—C1—O1	−0.22 (19)	C11—C12—C13—C14	−179.2 (2)
Cd1—O2—C1—C2	−178.62 (18)	C18—N2—C14—C15	1.2 (3)
Cd1—O1—C1—O2	0.3 (2)	Cd1—N2—C14—C15	175.77 (16)
Cd1^ii^—O1—C1—O2	155.28 (18)	C18—N2—C14—C13	−177.6 (2)
Cd1—O1—C1—C2	178.79 (13)	Cd1—N2—C14—C13	−3.1 (2)
Cd1^ii^—O1—C1—C2	−26.2 (3)	N1—C13—C14—N2	6.1 (3)
Cd1^ii^—O1—C1—Cd1	155.0 (3)	C12—C13—C14—N2	−174.6 (2)
O1—Cd1—C1—O2	−179.8 (2)	N1—C13—C14—C15	−172.8 (2)
O4^i^—Cd1—C1—O2	−107.13 (14)	C12—C13—C14—C15	6.5 (3)
N2—Cd1—C1—O2	81.94 (13)	N2—C14—C15—C16	−1.1 (3)
N1—Cd1—C1—O2	10.24 (14)	C13—C14—C15—C16	177.7 (2)
O1^ii^—Cd1—C1—O2	165.61 (13)	C14—C15—C16—C17	−0.2 (4)
O3^i^—Cd1—C1—O2	−82.28 (13)	C15—C16—C17—C18	1.2 (4)
C8^i^—Cd1—C1—O2	−91.92 (13)	C14—N2—C18—C17	−0.1 (4)
Cd1^ii^—Cd1—C1—O2	169.82 (14)	Cd1—N2—C18—C17	−174.43 (19)
O4^i^—Cd1—C1—O1	72.63 (16)	C16—C17—C18—N2	−1.1 (4)
N2—Cd1—C1—O1	−98.30 (13)		

Symmetry codes: (i) *x*+1, *y*, *z*; (ii) −*x*+1, −*y*, −*z*+1; (iii) *x*−1, *y*, *z*.

### Hydrogen-bond geometry (Å, °)

**Table:**

*D*—H···*A*	*D*—H	H···*A*	*D*···*A*	*D*—H···*A*
O1W—H1WA···O3^iv^	0.92 (2)	2.19 (4)	2.980 (4)	144 (5)
O1W—H1WA···O1W^v^	0.92 (2)	2.38 (6)	2.833 (8)	110 (5)

Symmetry codes: (iv) −*x*, −*y*, −*z*; (v) −*x*+1, −*y*, −*z*.
